# Whole-genome analysis of mRNA decay in *Plasmodium falciparum *reveals a global lengthening of mRNA half-life during the intra-erythrocytic development cycle

**DOI:** 10.1186/gb-2007-8-7-r134

**Published:** 2007-08-07

**Authors:** Jennifer L Shock, Kael F Fischer, Joseph L DeRisi

**Affiliations:** 1 Department of Biochemistry and Biophysics, University of California San Francisco, 1700 4^th ^Street, San Francisco, California 94158-2330, USA.; 2 Howard Hughes Medical Institute, Jones Bridge Road, Chevy Chase, Maryland 20815-6789, USA.

## Abstract

**Background:**

The rate of mRNA decay is an essential element of post-transcriptional regulation in all organisms. Previously, studies in several organisms found that the specific half-life of each mRNA is precisely related to its physiologic role, and plays an important role in determining levels of gene expression.

**Results:**

We used a genome-wide approach to characterize mRNA decay in *Plasmodium falciparum*. We found that, globally, rates of mRNA decay increase dramatically during the asexual intra-erythrocytic developmental cycle. During the ring stage of the cycle, the average mRNA half-life was 9.5 min, but this was extended to an average of 65 min during the late schizont stage of development. Thus, a major determinant of mRNA decay rate appears to be linked to the stage of intra-erythrocytic development. Furthermore, we found specific variations in decay patterns superimposed upon the dominant trend of progressive half-life lengthening. These variations in decay pattern were frequently enriched for genes with specific cellular functions or processes.

**Conclusion:**

Elucidation of *Plasmodium *mRNA decay rates provides a key element for deciphering mechanisms of genetic control in this parasite, by complementing and extending previous mRNA abundance studies. Our results indicate that progressive stage-dependent decreases in mRNA decay rate function are a major determinant of mRNA accumulation during the schizont stage of intra-erythrocytic development. This type of genome-wide change in mRNA decay rate has not been observed in any other organism to date, and indicates that post-transcriptional regulation may be the dominant mechanism of gene regulation in *P. falciparum*.

## Abstract 2

A consistent bias in tree reconciliation methods is described that occurs when the inferred gene tree is not correct, casting doubt on previous conclusions about ancient duplications and losses in vertebrate genome history.

## Background

*Plasmodium falciparum *is the most deadly of the four *Plasmodia *spp. that cause human malaria, and it is responsible for more than 500 million clinical episodes and 1 million deaths per year [1]. Because of increasing worldwide resistance to the most affordable and accessible antimalarial drugs, this number is expected to increase in the near future. In fact, deaths from malaria have increased over the past 6 years, despite a global health initiative designed to halve the burden of malaria by 2010 [2]. Gaining a more thorough understanding of the molecular biology of *P. falciparum *is an important step toward the identification of new drug and vaccine targets.

The *P. falciparum *48-hour asexual intra-erythrocytic development cycle (IDC) is characterized by the progression of the parasite through several distinct morphologic stages: ring, trophozoite, and schizont. Each cycle begins with invasion of an erythrocyte by a merozoite, followed by the remodeling of the host cell in the ring stage. The parasite then progresses into the trophozoite stage, where it continues to grow and is highly metabolically active. Finally, in the schizont stage, the parasite prepares for the next round of invasion by replicating its DNA and packaging merozoites.

The completion of the *P. falciparum *genome sequence represents a milestone in our understanding of this parasite and subsequently enabled numerous genomic and proteomic projects [3]. In previously reported work, our laboratory exhaustively profiled genome-wide mRNA abundance at a 1-hour time resolution throughout the IDC for three separate strains of *P. falciparum *[4,5]. Analysis of the IDC transcriptome revealed a cascade of highly periodic gene expression, unlike that seen in any other organism studied to date. Little is known about how this unique pattern of regulation is established or maintained.

The relative abundance of mRNA, as measured by conventional expression profiling, is a result of the rate at which each message is produced, offset by the rate at which each message is degraded. When compared with organisms with similar genome sizes, the *P. falciparum *genome appears to encode only about one-third the number of proteins associated with transcription [6]. Given this apparent lack of a full transcriptional control repertoire, unexpected post-transcriptional mechanisms, including mRNA decay, may contribute significantly to gene regulation.

Currently, very little is known about the components of mRNA decay in *P. falciparum*, and few of the proteins involved in mRNA decay are annotated. Using the protein sequence of known decay factors from humans and *Saccharomyces cerevisiae*, we identified putative orthologs to decay components (Table 1).

**Table 1:** Putative decay components in *Plasmodium falciparum *were identified using known factors from human and yeast

Components	Gene	PlasmoDB ID	Description
Deadenylation	Ccr4	PFE0980c^†^	Catalytic subunit of the Ccr4/Pop2 deadenylase complex
	Pop2	MAL8P1.104^†^	Component of the Ccr4/Pop2 deadenylase complex
	Not1	PF11_0049^†^	Component of the Ccr4/Not complex
	Not2	PF11_0297^†^	Component of the Ccr4/Not complex
	Not3	?	Component of the Ccr4/Not complex
	Not4	PFL1705w^†^	Component of the Ccr4/Not complex
	Not5	PF10_0062^†^	Component of the Ccr4/Not complex
	Caf130	?	Component of the Ccr4/Not complex
	Caf40	PFE0375w	Component of the Ccr4/Not complex
	PARN	PF14_0413*^†^	Major deadenylase in mammals
	Pab1	PFL1170w	PolyA binding protein
	Pan2	?	Component of the Pan2/Pan3 deadenylase complex
	Pan2	?	Component of the Pan2/Pan3 deadenylase complex
Decapping	Dcp1	PF10_0314*^†^	Component of the Dcp1/Dcp2 decapping complex
	Dcp2	Pf13_0048^†^	Catalytic subunit of the Dcp1/Dcp2 decapping complex
	DcpS/Dcs1	?	Scavenger decapping enzyme
	Dhh1	PFC0915w	Helicase-roles in deadenylation and decapping
	Lsm1	PF11_0255^†^	Involved in decapping
	Lsm2	PFE1020w	Involved in decapping
	Lsm3	PF08_0049	Involved in decapping
	Lsm4	PF11_0524	Involved in decapping
	Lsm5	PF14_0411	Involved in decapping
	Lsm6	PF13_0142	Involved in decapping
	Lsm7	PFL0460w	Involved in decapping
Exosome	Csl4	MAL7P1.104	Exosome subunit
	Dis3/Rrp44	MAL13P1.289^†^	Exosome subunit-RNase II domain
	Mtr3	?	Exosome subunit-RNase PH domain
	Rrp4	PFD0515w	Exosome subunit-hydrolytic exonuclease
	Rrp40	MAL13P1.36^†^	exosome subunit-hydrolytic exonuclease
	Rrp42	MAL13P1.204^†^	Exosome subunit-RNase PH domain
	Rrp43	?	Exosome subunit-RNase PH domain
	Rrp45	PF13_0340	Exosome subunit-RNase PH domain
	Rrp46	?	Exosome subunit-RNase PH domain
	Rrp6	PF14_0473	Exosome subunit found only in the nucleus
	Ski2	PFI0480w	Helicase associated with the exosome and Ski complex
	Ski3/Ski5	?	Associated with exosome and Ski complex
	Ski6/Rrp41	PF14_0256	Exosome subunit-RNase PH domain
	Ski7	?	Associated with exosome and Ski complex
	Ski8	?	Associated with exosome and Ski complex
5' to 3' decay	Xrn1	PFI0455w/PF11_0074^†^	5' to 3' exonuclease-cytoplasmic
	Rat1	PFI0455w/PF11_0074^†^	5' to 3' exonuclease-nuclear

Orthologs were identified using a simple reciprocal best BLASTP match between *Plasmodium falciparum *and *Saccharomyces cerevisiae *and between *P. falciparum *and human sequences. Orthologs could not be found for those genes with question marks. *Components identified only when the human sequence was used for the query sequence. ^†^*P. falciparum *proteins that either are described in PlasmoDB as hypothetical proteins or, in the case of PF10_0314, are assigned a function other than the one relevant here.

Studies in mammals and the budding yeast *S. cerevisiae *have identified two major pathways for the degradation of mRNA, both of which are deadenylation dependent: 5' to 3' decay and 3' to 5' decay [7]. Both pathways of mRNA decay in mammals and *S. cerevisiae *begin with deadenylation, which is carried out by 3' exonucleases specific for the poly(A) tail, and is thought to be the rate-limiting step of mRNA decay [8]. The major deadenylase in mammalian cells is PARN (poly(A) specific ribonuclease), an RNase D homolog that is conserved in many eukaryotes. In *S. cerevisiae*, deadenylation is carried out by two complexes: Pan2p/Pan3p and Ccr4p/Pop2p. The Ccr4p/Pop2p complex is the dominant deadenylase in yeast, and is part of a much larger complex called the Ccr4/Not complex, which also plays roles in transcription initiation and elongation, and protein modification, suggesting that these processes may be coordinately regulated [9,10].

Following deadenylation, transcripts can be degraded via one of two distinct pathways. In 5' to 3' decay Dcp1p and Dcp2p decap the transcript, leaving the 5' end vulnerable to decay. There are also a number of enhancers and regulators of decapping, including Edc1p-3p, the Lsm complex, and the DEAD box helicase Dhh1p. After decapping, subsequent degradation of the transcript proceeds through Xrn1p, the major 5' to 3' exonuclease. Eukaryotes also possess a related exonuclease called Rat1p, which is thought to function mainly in the nucleus.

In contrast to 5' to 3' decay, in 3' to 5' decay transcripts are degraded by the exosome following deadenylation. The exosome is a complex containing ten essential proteins, nine of which have a 3' to 5' exonuclease domain. An 11th component, Rrp6p, is associated with the exosome only in the nucleus and is not essential. Also associated with the exosome are two helicases: Ski2p and Mtr4p. Finally, the free cap generated by 3' to 5' decay is hydrolyzed by the scavenger protein Dcs1p.

Orthologs for Rrp43p, Rrp46p, and Mtr3p have not yet been identified in *P. falciparum*. Interestingly, these same proteins were also difficult to identify in *Trypanosoma brucei*, but they were eventually identified through association with other exosome components [11,12]. Unfortunately, using the newly identified *T. brucei *components does not identify any additional exosome components in *P. falciparum*.

The numerous factors that constitute the decay machinery regulate the degradation rates of every cellular transcript. The complexity of the system allows for many possible avenues of regulation, including interactions between specific RNA binding proteins and motifs in the 3' untranslated regions (UTRs) of transcripts, developmental regulation of decay components, and localization of factors or RNAs to specific compartments in the cell such as P bodies [13]. Any of these methods of regulation are possible in *P. falciparum*. In particular, developmental regulation of decay components is an attractive model, considering that many of the putative decay components in *P. falciparum *are transcriptionally regulated during the IDC, with peak expression during the ring to early trophozoite stages [4].

In mammals and yeast, the specific half-life of each mRNA is precisely related to its physiologic role and in many cases can be altered in response to different stimuli or developmental conditions [13-16]. For example, it was found in yeast that core metabolic genes such as those encoding glycolytic enzymes produce mRNAs with very long half-lives, but genes encoding components of the mating type signaling pathway produce mRNAs with very short half-lives.

Thus far, decay rates have not been determined for any *P. falciparum *transcripts, and neither have any of the mRNA decay components been genetically or biochemically characterized. Given that mRNA decay is an integral component of gene expression regulation, we conducted a genome-wide study of mRNA decay in *P. falciparum *using a microarray-based approach to measure mRNA half-life as a function of the IDC. Interestingly, we found that a major determinant of mRNA decay rate appears to be tightly linked to IDC, and to a lesser extent the functional category of the mRNAs themselves. Decay rates in the early hours after invasion are rapid and tightly distributed, but by the end of the cycle global decay rates decrease considerably, causing a lengthening of half-lives. An analogous genome-wide change in mRNA decay rate during a development cycle has not been observed in any other organism to date.

## Results

### Overview of the data

**Figure 1:**
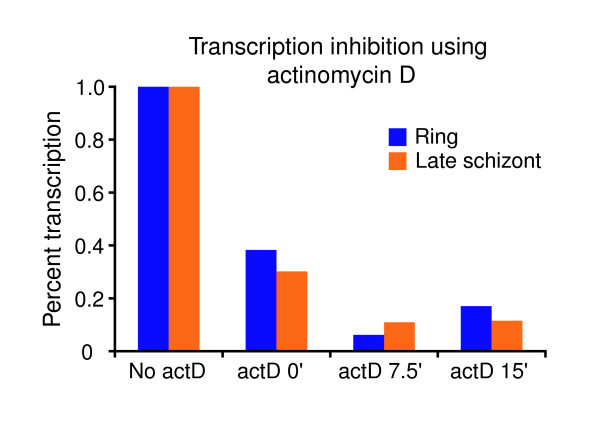
Nuclear run-on analysis shows that actD halts transcription in *Plasmodium falciparum*. Actinomycin D (actD) was added to synchronous cultures in the ring and late schizont stages. Time points were then taken before addition of actD and then at 0, 7.5, and 15 min intervals after addition of drug. The samples were normalized such that the no actD sample was normalized to 100% transcription.

To explore the role of mRNA decay during the IDC of *P. falciparum*, we used microarrays to determine decay rates on a genome-wide scale at four distinct stages. Using aliquots from a single synchronized culture of 3D7 strain parasites (sequenced strain), transcription was inhibited and total RNA was harvested for microarray hybridization. Transcriptional shut off was achieved by addition of actinomycin D (actD), which is known to intercalate into DNA and inhibit DNA-dependant RNA polymerases [17]. ActD has also been shown previously to inhibit *P. falciparum *transcription strongly in a dose-dependent manner, with little or no RNA synthesis seen at higher drug concentrations [18]. We further confirmed transcription inhibition in our own experimental conditions through nuclear run-on experiments using synchronous cultures in the ring and late schizont stages, and we note that the degree of relative transcriptional inhibition was approximately equal between ring and schizont stage parasites (Figure 1). Although some residual labeling of nuclear run-on RNA was observed after treatment with actD in both stages, any remaining transcriptional activity over the course of the experiment is not anticipated to affect the determination of the decay rate (see Materials and methods, below). Although it remains a formal possibility that treatment with actD could alter the activity of decay processes through indirect or secondary effects, no such effects have been reported in other organisms to our knowledge.

**Figure 2:**
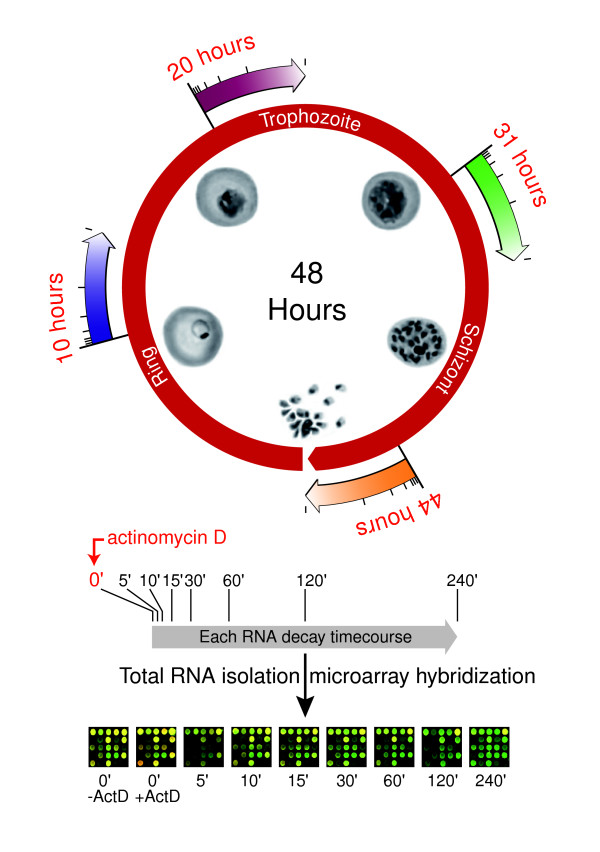
Schematic of the microarray experiment to determine half-lives through the life cycle. Four separate time course experiments were conducted at 12-hour intervals using a single source culture of synchronized parasites. Numbers in red represent the hour after invasion when actD was added in relationship to the previously published transcriptome experiment. Total RNA was subsequently harvested at the indicated time points. These samples were reverse transcribed into cDNA and hybridized to DNA microarrays. Specific spiked controls were included to determine correct normalization during microarray scanning.

For each of the four decay time courses, an initial sample was harvested immediately before addition of drug, followed by eight more samples at intervals from 0 to 240 min (Figure 2). Each sample was mixed with a reference pool and applied to a 70 mer DNA oligonucleotide microarray in a standard two-color competitive hybridization [19]. This experiment was repeated every 12 hours throughout the IDC, starting in the ring stage. A Pearson correlation was used to compare the zero minute (untreated cells) results at each of the four stages with the previously characterized 48-hour transcriptome of the HB3 strain [4]. The highest correlations between the decay experiments and the transcriptome data were at 10, 20, 31, and 44 hours after invasion, corresponding to the ring, trophozoite, schizont, and late schizont stages, respectively (*r *= 0.79, 0.80, 0.67, and 0.73, respectively). The hours of peak correlation for subsequent time points in each of the four separate time courses were unchanged, although the actual correlation value progressively decreased, consistent with global transcriptional shutoff at each stage. Using RNA samples from the same experiment, each microarray hybridization was performed at least twice, and in most cases three times.

**Figure 3:**
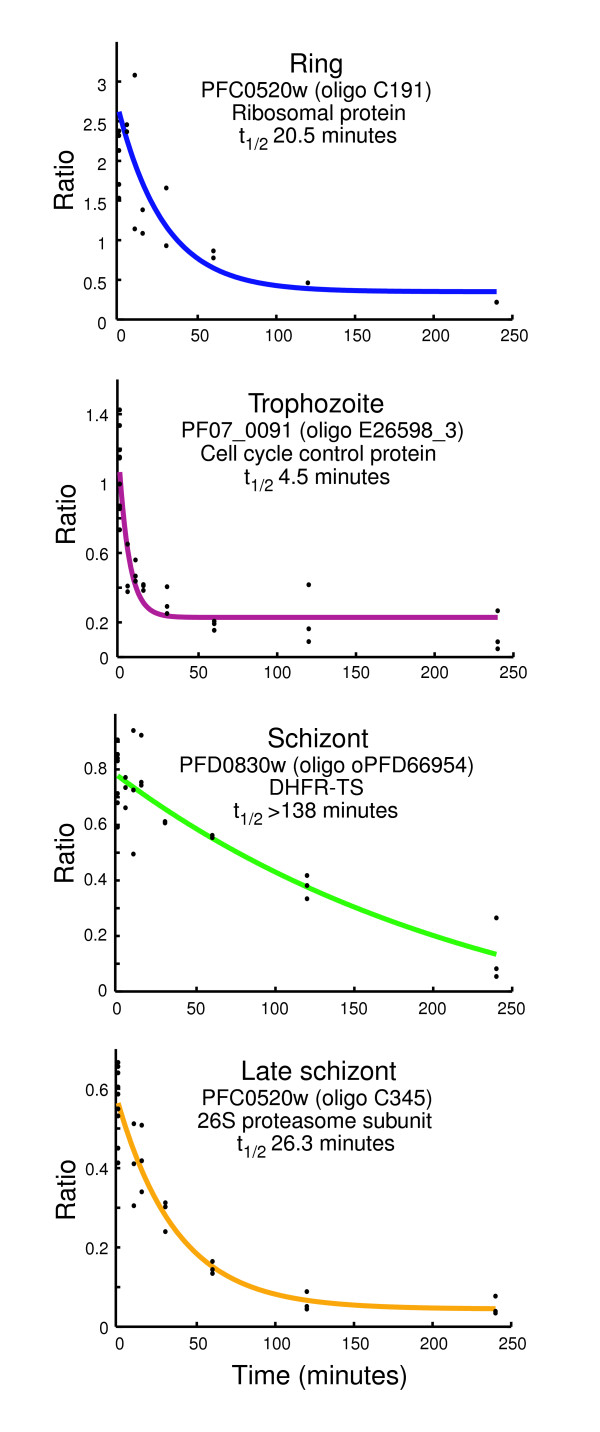
Examples of mRNA decay profiles for each stage determined by microarray analysis. Four example genes were chosen to demonstrate the range of half-lives that can be measured in this experiment. The black dots represent data points from each of the microarray replicates for that time point, including the 0 time point with and without actinomycin D treatment. The colored lines represent the fitted decay curve. The half-life (t_1/2_) for each example is listed.

The data from each series of microarrays were fit to an exponential decay curve using nonlinear least squares fit, and the half-life was calculated for the transcript hybridized to each oligo at each of the four stages analyzed (Additional data file 1). Figure 3 shows arbitrarily chosen examples of decay curves for four individual transcripts, each at one of the four stages. A set of 6,225 oligos (representing 4,783 genes) passed our quality control filters and had a fit decay curve for at least one stage (see Materials and methods, below). Of these, 3,903 oligos (representing 2,744 genes) had a calculated half-life for all four stages. The decay curves for these genes are available at the DeRisi laboratory malaria database [20]. Those oligos that did not pass the quality control filters are listed in Additional data file 2.

Calculated half-lives ranged from 1 min to longer than 138 min, which was the maximum half-life that could be reliably fit given that each time course was terminated at 240 min after addition of actD. As previously noted, we cannot rule out low levels of ongoing transcription after the addition of drug, but an ongoing zero-order process would not affect our fitted half-lives (see Materials and methods, below). Because many genes are represented by more than one oligo, it is possible to use this information to corroborate internally the microarray measurement and calculation of half-lives. In general, half-lives for oligos within a single open reading frame (ORF) agreed well (Table 2). For example, the average standard deviation for half-lives in the ring stage was 9.9 min, whereas the average standard deviation for half-lives of oligos within a single ORF in the ring stage was 4.6 min. Depending on the stage, 13% to 17% of genes that have more than one oligo had poor intragenic correlations, with greater than two standard deviation difference for oligos within a single ORF. There may be underlying technical or biologic explanations for discrepancies in these genes. Because the vast majority of annotated genes in the *P. falciparum *genome exist only as gene models without experimental validation, oligos thought to be common to a given gene may in fact be hybridizing to distinct transcripts. Furthermore, alternative splicing of transcripts and bias in the directionality of decay may also result in poor intragenic correlations.

**Table 2:** Average half-lives and standard deviations for each stage

Stage	Half-life (min)	Standard deviation (min)	Standard deviation for oligos within a single ORF
Ring	9.5	9.9	4.6
Trophozoite	20.5	28.5	9.9
Schizont	49.9	37.3	21.9
Late schizont	65.4	42.6	22.2

ORF, open reading frame

### All mRNA half-lives increase during the intra-erythrocytic development cycle

**Figure 4:**
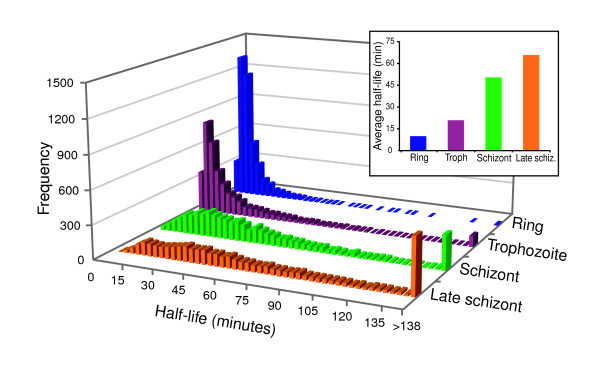
The distribution of mRNA half-lives changes for each stage of erythrocytic development. Both the histogram and the graph of mean half-lives for each stage (inset) reveal that half-lives increase on a global scale over the course of the intra-erythrocytic development cycle.

Figure 4 shows the distributions of mRNA half-lives for each stage. Beginning with ring stage parasites and ending with late schizonts, we observed a striking increase in half-life for essentially all transcripts measured as a function of the IDC. In the ring stage the mean half-life was 9.5 min and the distribution of half-lives was very narrow, with a standard deviation of only 9.9 min (Table 2). In the later stages, the average half-life progressively lengthened and the distribution progressively widened. By the late schizont stage, the mean half-life for all transcripts had increased more than sixfold (65.4 min) and the standard deviation had increased by fourfold (42.6 min). Although the scope of mRNA decay studies has been limited to model systems or mammalian cells, this progressive and dramatic shift in global mRNA decay rates as a function of developmental cell cycle has not previously been observed in any other organism to date.

We compared half-life with ORF length and relative transcript abundance and, as in yeast and mammalian cells, no strong correlation was found. This indicates that mRNA decay in *P. falciparum *is most likely a regulated process rather than a simple, basal degradation of all transcripts [14,21]. Although there exists a global trend in decay rate change that is common to all genes, we sought to determine whether individual patterns of rate change correlated with the corresponding profile of steady-state abundance, measured previously [5]. We found no significant correlation between the transcriptome phase of each gene (roughly, when the peak of expression occurs during the IDC) and its pattern of half-life change (data not shown).

**Figure 5:**
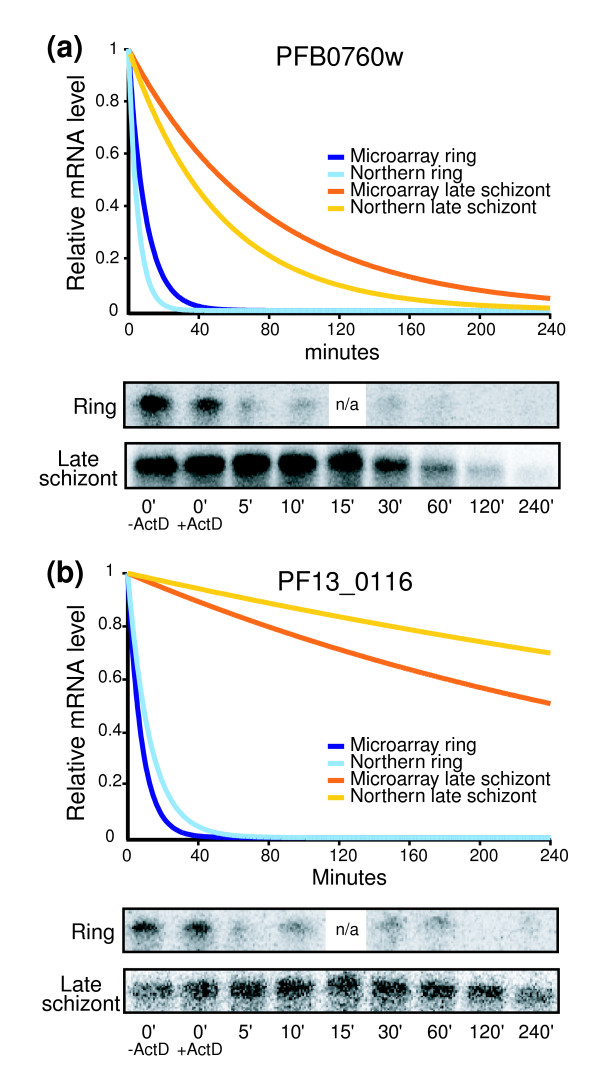
Comparison of decay rate calculated by microarray and by Northern blot. The half-lives for **(a) **PFB0760w and **(b) **PF13_0116 were verified by Northern blot analysis (quantified by PhosphorImager) for the ring and late schizont stages using total RNA from the same experiment. All of the microarray replicates were used to calculate the decay rate from the microarrays.

The half-lives for PFB0760w and PF13_0116, two genes with large stage-dependent changes in decay rate, were confirmed by Northern blot analysis for the ring and late schizont stages (Figure 5). Half-lives calculated by Northern blot were in good agreement with those calculated by microarray. The half-lives for PFB0760w in the ring and schizont stages were calculated to be 6.7 and 54.1 min, respectively, by microarray analysis and 3.7 and 35.6 min by Northern analysis. For PF13_0116 the half-life calculated by microarray was 5.4 min in the ring stage, as compared with 8.8 min calculated by Northern blot. The half-life for the late schizont stage was greater than 138 min when calculated using either method.

It has previously been shown that transcripts from *Plasmodium *spp. can have multiple polyadenylation sites [22,23]. Although no obvious difference in transcript length was evident at the resolution of the Northern blots, we specifically investigated the possibility that changes in UTR length were concomitant with changes in decay rate. We analyzed the 5'-UTRs of PF13_0116 and PFB0760w (the same genes used in the Northern blot analysis) using rapid amplification of cDNA ends (RACE) for the ring and late schizont stages. The lengths of the 5'-UTRs for these two genes were measured to be 185 base pairs (bp) and 836 bp, respectively. For both genes in both stages there was no change in the RACE products and, at least for these representative genes, changes in site selection for transcription initiation were not evident and are therefore not linked to the observed lengthening of half-life during the IDC. We attempted 3'-RACE on these genes, but because of the extreme A/T richness of *P. falciparum *UTRs, we cannot be certain that the UTRS were accurately measured. Thus, a change in transcription termination or polyadenylation site could be linked to the lengthening of half-lives.

### Decay rate and gene function

**Figure 6:**
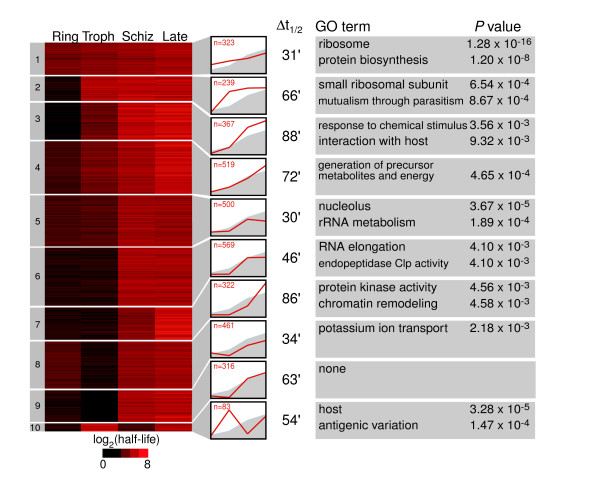
*K*-means clusters of the half-life data for each stage. Genes were clustered into 10 *k*-means clusters using the log_2 _transformed half-life (t_1/2_; minutes) in each stage. The average half-life was used for genes represented by more than one oligo (see Materials and methods). In the plot to the right of each *k*-means cluster, the average decay profile for each group is displayed (red line) with the average decay profile for the entire dataset (gray line filled down). The *x*-axis represents the four stages progressing through the life cycle from rings to late schizonts. The *y*-axis represents half-life from 0 to 100 min. Δt_1/2 _represents the average half-life difference between the late schizont stage and the ring stage for that group (late schizont half-life - ring half-life). On the right are the top two most significant Gene Ontology terms for each *k*-means cluster (GoStat was used for this analysis).

In yeast and mammalian cells there is a relationship between decay rate and gene function [14-16]. In particular, transcripts for proteins that function in the same pathway or process generally have similar decay rates. Because the rate of decay for most *P. falciparum *transcripts changes during the IDC, it is inappropriate to determine a single half-life for each transcript. However, we wished to investigate the possibility that the pattern of decay rate change could be used to partition the dataset into distinct groups. We used *k*-means clustering of the half-lives for all four stages into ten groups followed by Gene Ontology (GO) term analysis (GoStat) to detect enrichment of gene function or process (Figure 6) [24]. We tested several different numbers of groups, and dividing the data into ten groups best matches the natural structure of the data, and gives the lowest *P *values for the GO term analysis. To ensure that the clusters were not an artifact of the cyclic nature of steady-state expression *in P. falciparum*, we compared the distributions of transcriptome phases, which represent the stage of peak expression, for each group and found no significant correlation (data not shown).

Although all ten groups have a pattern of increasing half-lives in which the late stage half-lives are longer than the ring stage half-lives, there are differences in the pattern and scale of half-life increase. These differences are illustrated in the plots showing the average half-lives for each group and the increase in half-life from the ring to late schizont stage in Figure 6. The decay rate progressively decreases in each stage for six of the ten groups. Of the four remaining groups, groups 8 and 9 have trophozoite stage half-lives that are shorter than their ring stage half-lives, group 5 has late stage half-lives shorter than its schizont stage half-lives, and group 10 has a unique decay pattern with transcripts being most stable in the trophozoite stage.

Nine of the ten groups are associated with significantly over-represented GO terms, and the complete list of significant terms and corresponding *P *values can be found in Additional data file 3. A range of GO terms was represented among the different groups, and in general over-represented terms in the same group were involved in similar processes. Listed below are the four groups with the most significant GO terms (lowest *P *value).

Transcripts clustered in group 1 exhibit a relatively stable pattern of decay, with an increase in half-life of 31 min between the ring and late schizont stages, as compared with a genome-wide average increase of 56 min. Group 1 also has the longest average half-life in the ring stage. This group has several highly over-represented terms, including ribosome, ribonucleoprotein complex, cytoskeleton organization and biogenesis and mitochondrion. Given that all of these terms represent proteins involved in processes that are active throughout the life cycle, it is not surprising to see them in the cluster with the lowest half-life variability across the IDC. Although group 4 also has over-represented GO terms for processes needed throughout the life cycle, the genes in this group have a pattern of decay that most closely matches the average half-life increase seen across the genome. Terms for the generation of metabolites and energy, cellular catabolism, and tricarboxylic acid cycle metabolism were all found in this group. Wang and colleagues [14] found that, in yeast, genes involved in energy metabolism had similar, very long half-lives. Instead, our data suggest that the rate of decay in *P. falciparum *might be matched to the energy requirements of the growing cell. Group 5 also has a relatively stable pattern of decay, but it does not have longer than average half-lives in the ring stage. This group is enriched in terms involved in RNA processing, including nucleolus, rRNA metabolism, and nuclear mRNA splicing, via spliceosome. Group 10 has the most unusual profile, with a short average half-life in the ring and schizont stages and a long average half-life in the trophozoite and late schizont stages. This group has terms involved in pathogenesis overrepresented, such as host and antigenic variation.

In a separate analysis, we were able to find over-representation for two sample groups that could be easily annotated by hand using the lexical analysis tool LACK [25]. Plastid encoded genes and tRNAs, which both lack associated GO terms, were over-represented in group 7 (*P *< 3.25 × 10^-3 ^and 4.50 × 10^-5^, respectively).

## Discussion

In this study we showed that the overall rates of decay change during the developmental cycle, beginning with relatively short half-lives in the ring stage and ultimately ending with dramatically longer half-lives in the schizont stage. The abundance of any given mRNA species is the result of transcriptional production offset by the rate of degradation. Thus, a change in transcriptional output without a corresponding change in abundance implies a necessary alteration in the rate of degradation. It has previously been noted that the quantity of mRNA that can be isolated per infected erythrocyte increases dramatically over the course of the IDC [26]. There is also evidence, through previously reported radioisotope pulse labeling experiments and our own unpublished observations, that transcription increases steadily from invasion until around 36 hours and then drops off in late stage parasites [27,28]. Therefore, the observed mRNA accumulation after schizogony appears to be largely a function of enhanced mRNA stability rather than increasing levels of transcription.

This global stabilization may be a mechanism to regulate gene expression in late stage parasites, when the process of packaging multiple nuclei into developing merozoites may complicate coordinate regulation of transcription. Stabilized mRNA may also be important for the merozoite, and represent a carefully regulated 'starting package' that would allow rapid activation of the IDC following the next round of invasion. Furthermore, the data are consistent with a process by which the low level transcriptional accumulation in early stage parasite development features rapid mRNA turnover, perhaps indicative of the dynamic remodeling process that immediately follows invasion. Future experiments using a pulse chase system, such as was developed for *Toxoplasma gondii *to measure newly synthesized mRNA and its degradation, could also help to elucidate whether this model is correct [29].

The mechanism for the genome-wide increase in mRNA half-lives remains unclear. Similar to yeast and mammalian cells, the rate of decay could not be correlated to transcript length, abundance, or transcriptome phase [14,21].

A straightforward mechanism that could explain the global lengthening of half-lives would include the developmental regulation of the decay components themselves. Indeed, all of the decay components measured by our previous transcriptome profiling efforts exhibit clear patterns of phase-specific expression, with most profiles indicating peak mRNA abundance in rings and trophozoites [4]. Although the mRNA abundance profiles of the decay components are consistent with a model in which the most rapid turnover of mRNA occurs early in the IDC, the actual protein expression profiles of these components remain to be measured. Furthermore, the expression of the proteins themselves may not be coordinated with their activation, and so the actual biochemical activities must also be assessed. For example, a progressive decrease in deadenylation, decapping, or exonuclease activity could account for the observed lengthening of half-lives independent of when the proteins are produced. Given the current data, it remains an open question as to whether the observed increases in mRNA half-life are a direct result of limiting quantities of critical decay components or whether additional regulatory factors or physical sequestration limit entry of mRNAs into the decay pathway.

Numerous studies in other organisms have shown that sequence elements in the 3'-UTR are important in determining decay rate for many genes [10]. The extreme A/T richness of the *P. falciparum *genome, combined with a lack of functionally characterized UTRs, has made identification of putative decay motifs, which are also generally A/T rich, rather challenging. However, Coulson and coworkers [6] found proteins with sequence similarity to RNA binding proteins, in particular the CCCH-type zinc finger proteins that are involved in regulating mRNA stability, to be over-represented in *P. falciparum*.

In addition to the global change in decay rate, we also showed that genes grouped by their pattern of decay exhibit significant enrichment of GO terms, suggesting that genes functioning in the same pathway or process have similar decay rates over the life cycle. This type of coherence in mRNA decay rates for functional groups has also been observed in yeast and mammals [14,16], and at least two separate factors - RNA polymerase II subunit Rpb4p and Pub1p - have been linked to differential decay of mRNAs encoding protein biogenesis components [30,31]. In their study of changes in transcription rate and decay rate during the shift from glucose to galactose in yeast, Garcia-Martinez and colleagues [16] identified several GO categories that have coordinately regulated decay patterns. Interestingly, many of these categories are the same as those identified in this study, including ribosomal proteins, proteins involved in energy metabolism, and proteins involved in rRNA processing. These authors also measured a general increase in mRNA stability during the shift from glucose to galactose. An analogous mechanism may be responsible for the observed increase in mRNA stability during the *P. falciparum *developmental cycle.

## Conclusion

In this study we have measured the mRNA decay rate in *P. falciparum *for more than 4,000 genes during the 48-hour intra-erythrocytic life cycle. The characterization of mRNA decay rates on a genome-wide scale during the *P. falciparum *IDC offers insights into the unique biology of the malaria parasite and the unique manner in which gene expression is regulated throughout the IDC. This study provides a foundation for continued investigations into the molecular mechanism of *Plasmodium *mRNA decay and its role in parasite development.

## Materials and methods

### Cell culture

*P. falciparum *parasite cells (3D7) were cultured as described previously [19]. Cells were synchronized by two consecutive sorbitol treatments during two consecutive cell cycles (a total of four treatments), and the mRNA decay experiments were conducted at 12-hour intervals starting in the ring stage (10 to 12 hours after invasion). To stop transcription actD (Amersham, Piscataway, NJ, USA) was added at 20 μg/ml, and samples were collected 0, 5, 10 15, 30, 60, 120, and 240 min later. For each mRNA decay experiment, a sample was also taken directly before addition of drug. The cells were harvested by centrifugation at 1,500 *g *for 5 min, washed in phosphate-buffered saline (PBS), and pelleted at 1,500 *g *for 5 min. Cell pellets were rapidly frozen in liquid nitrogen and stored at -80°C.

### RNA preparation and microarray hybridization

Total RNA was prepared directly from the frozen pellets of parasitized erythrocytes, in which approximately 1 ml of cell pellet was lysed in 10 ml Trizol (Invitrogen, Carlsbad, CA, USA). For the hybridization experiments, 8 μg total RNA was used for amino-allyl cDNA synthesis, as previously described [19]. A pool of amino-allyl labeled cDNAs representing stages throughout the IDC was assembled and used as a reference. For DNA microarray hybridization, the pool cDNA was always coupled to Cy3 dye as reference, whereas cDNA from an individual time point was coupled to Cy5 dye.

The DNA microarray used in this study contains 8,182 70-mer oligos. Of these, 6,652 are unique and map to an annotated ORF, as listed in PlasmoDB release 4.4. DNA microarrays were printed and postprocessed as described previously [19].

For the trophozoite, schizont, and late schizont decay experiments, the individual time points were hybridized in triplicate. For the ring decay experiment, the time points were hybridized in duplicate because of limiting quantities of RNA. In all cases, all time points for a single replicate were hybridized on the same day and included two replicates of the sample collected before addition of actD.

DNA microarrays were scanned using an Axon 4000B scanner and images analyzed using Axon GenePix software (Molecular Devices, Union City, CA, USA). Microarray data were stored using NOMAD database software [32]. All microarray data are available at the Gene Expression Omnibus [33] (series accession number GSE8099).

For normalization among time points, an internal control was prepared with a pool of *in vitro *transcribed *S. cerevisiae *RNAs. Oligos Y_IBX1991, Y_ICX1881, Y_IFX1541, and Y_IHX3161, representing four intergenic *S. cerevisiae *sequences, were printed 16 times each onto the *P. falciparum *DNA microarrays [34]. RNA transcripts of each of these *S. cerevisiae *DNAs were prepared *in vitro *and pooled. This internal control mix was added to each total RNA sample analyzed in the decay time course, as well as to the total RNA used as pool, at a final concentration of 1 ng of each *S. cerevisiae *RNA per 8 μg of total RNA.

### Data analysis

Decay rates for every transcript for which decay was observed were determined by fitting all observations with a three-parameter first order decay model, A = (A_0 _- B)e^-ln(0.5)/t1/2 × t ^+ B, using the Levenberg-Marquardt algorithm implemented in MINPACK [35]. Using this model, fits of half-life (t_1/2_) are insensitive to zero order kinetic contributions, such as continued low levels of transcription. Formally, a zero order contribution to a first order process affects only the final steady state ratio, B in our model, and has no impact on the decay constant or half-life. To ensure that contributions from residual transcription were minimized, all measurements for times under 10 min were excluded from the analysis if no decay had been observed for the transcript in question.

All arrays were normalized using the average median intensities of the *in vitro *transcribed *S. cerevisiae *RNA internal controls. The ratio of median intensities for unflagged spots with a median intensity of greater than 300 in either channel was used for further analysis. Several quality control checks were employed before any fits were included in downstream analysis, as summarized in Additional data file 2. Each decay profile had to pass two sets of filters. First, before any fitting was performed, a set of raw data filters (group A; Additional data file 2) was applied. The profile had to include measurements from at least eight data points, the observed amplitude had to be at least 0.1, the first time point not later than 10 min, and the last observation not earlier than 60 min. Second, the decay profiles that passed all of the group A tests were fit to the decay model. Fits with systematic residuals were eliminated by filtering out fits in several classes (group B; Additional data file 2). Each fit had to have an amplitude (A_0 _- B) of at least 0.1 but not more than 10.0, with B between -1.0 and A_0_, and the fit half-life had to be greater than 1 min. When under 70% of the fit amplitude was experimentally observed over the time course, we report the half-life as greater than 138 min.

Oligos that were excluded from the final analysis are listed for each stage in Additional data file 2. Values for each model parameter, initial ratio (A_0_), final ratio (B), and t_1/2_, as well as the asymptotic standard errors for all fits that satisfy those criteria, are given in Additional data file 1.

*K*-means clusters were generated using log-transformed half-lives for each gene in each stage. For genes with more than one oligo, the half-lives were averaged if the standard deviation was less than the average standard deviation for all genes with multiple oligos in that stage. The clustering was done only on genes with data present for at least three out of the four stages. *K*-means clusters were generated using uncentered mean correlation in Cluster 3.0 and visualized using Java Treeview [36]. GO analysis was done using the GoStat tool with the following parameters: minimal length of considered GO paths = 5, maximal *P *value = 0.01, cluster GOs = -1, and correct for multiple testing = none.

### Nuclear run-on analysis

Synchronous cultures in either the ring or late schizont stage were treated with 20 μg/ml actD. Time points were then taken at 0, 7.5, and 15 min after addition of drug. Each sample was pelleted and washed once with 1 × PBS before lysis with 1 volume 0.2% saponin. Lysed parasites were washed twice in 1 × PBS and resuspended in 1.5 ml buffer A (20 mmol/l PIPES, 15 mmol/l NaCl, 60 mmol/l KCl, 14 mmol/l 2-mercaptoethanol, 0.5 mmol/l EGTA, 4 mmol/l EDTA, 0.5 mmol/l spermidine, and 0.125 mmol/l PMSF). The parasites were transferred to a Dounce homogenizer with a type B pestle. A volume of 100 μl 10% NP-40 was added and ten strokes were applied. Nuclei were pelleted by centrifugation for 3 min at 6,000 *g*, washed in buffer A, and pelleted again. Each sample was resuspended in 200 μl buffer B (50 mmol/l HEPES [pH 7.9], 50 mmol/l NaCl, 10 mmol/l MgCl_2 _1.2 mmol/l DTT, 1 mmol/l GTP, 1 mmol/l CTP, 4 mmol/l ATP, 20% glycerol, 25 U/mL RNasin). Then, 0.5 μmol/l [α-^32^P]UTP (3000 Ci/mmol) was added to all samples and transcription was allowed to proceed at 37°C for 30 min. RNA was isolated using Trizol, as per the manufacturer's instructions. The RNA was hybridized to a dot blot spotted with 500 ng total cDNA in Rapid Hyb buffer (Amersham) at 55°C and washed in accordance with the manufacturer's instructions. Spot intensity was measured using a Storm PhosphorImager (GE Healthcare Biosciences, Piscataway, NJ, USA).

### Rapid amplification of cDNA ends

5'-RACE was done using the First Choice RLM-RACE kit (Ambion, Austin, TX, USA), in accordance with the manufacturer's instructions. Nested polymerase chain reaction (PCR) was used and the following primers were employed with the primers provided with the kit: 5'-TGATTTTACGCTTAAACCAGAGG-3' for the PFB0760w 5'-RACE outer primer; 5'-CTCTTGTTACTATTATTATTTTGCCCCTCA-3' for the PFB0760w 5'-RACE inner primer; 5'-TGACCAAAAAGATTTTACTGAA-3' for the PF13_0116 5'-RACE outer primer; 5'-AAAACTTTCCAAACTTTCACAA-3' for the PF13_0116 5'-RACE inner primer. Herculase (Stratagene, La Jolla, CA, USA) was used for all PCRs using the following cycling conditions: 3 min at 94°C followed by 35 rounds of 94°C for 30 s, 55°C for 30 s, and 60°C for 1 min. All PCR products were confirmed by sequencing.

### Northern analysis

Northern blots were performed as described previously [37]. Total RNA (10 μg) was used for each sample. The probes were generated using the following PCR primers: 5'-CCAAAGGAGGAGACATCCAA-3' and 5'-GGGAAACACAATCGCTGAAT-3' for PFB0760w, and 5'-AGAATGCTTTCCCACGACAC-3' and 5'-TGAATCGTTAAAAGACGGATGA-3' for PF13_0116. The PCR products were labeled with [α-^32^P]dATP using the DECAprime™ II kit (Ambion). The RNA used for the Northerns was the same as that used for the cDNA synthesis.

## Additional data files

The following additional data are available with the online version of this paper. Additional date file 1 provides the complete dataset, containing initial ratio (A_0_), final ratio (B), and half-life (t_1/2_), as well as the asymptotic standard errors for each of these parameters. Additional data file 1 provides The complete dataset containing a list of oligos that did not pass the quality control filters in each stage. Additional data file 1 provides a complete list of all of the significant GO terms and associated *P *values found in each of the *k*-means clusters.

## Supplementary Material

Additional data file 1 Presented is the complete dataset containing initial ratio (A_0_), final ratio (B) and half-life (t_1/2_) as well as the asymptotic standard errors for each of these parameters. Click here for file

Additional data file 2 Presented is the complete dataset containing a list of oligos that did not pass the quality control filters in each stage. The specific filter or filters these oligos did not pass are listed. Click here for file

Additional data file 3 Presented is a complete list of all the significant Gene Ontology terms and associated *P *values found in each of the k-means clusters. Click here for file
